# Radicular cyst in a primary molar following pulp therapy with gutta percha: A case report and literature review

**DOI:** 10.4317/jced.55309

**Published:** 2019-01-01

**Authors:** Vo Truong-Nhu-Ngoc, Nguyen Vu-Thai-Lien, Luong Minh-Hang, Doan Thanh-Tung, Vu Thi-Nga, Hai Pham-The, Dinh-Toi Chu

**Affiliations:** 1 School of Dentistry, Hanoi Medical University, Hanoi, Vietnam; 2 Institute for Research and Development, Duy Tan University, 03 Quang Trung, Danang, Vietnam; 3 Hanoi University of Pharmacy, 13-15 Le Thanh Tong, Hoan Kiem, Hanoi, Vietnam; 4 Faculty of Biology, Hanoi National University of Education, Hanoi, Vietnam; 5 Centre for Molecular Medicine Norway (NCMM), Nordic EMBL Partnership, University of Oslo and Oslo University Hospital, Norway

## Abstract

A radicular cyst (RC) in deciduous dentition is relatively rare. This clinical report presents a case of RC that condition derived from a primary molar undergone an endodontic treatment with gutta-percha approximately one year ago. In addition, we also considered whether intracanal medicaments and gutta-percha filling material related to the formation and development of the cyst or not.

** Key words:**Primary tooth, radicular cyst, pulp therapy, gutta-percha filling material, intracanal medicament.

## Introduction

The radicular cyst (RC) is known as a common type of odontogenic cyst arising from the proliferation of the Hertwig’s epithelial root sheath (also called epithelial cell rests of Malassez - ERM). The RC is stimulated by a condition of pulpal necrosis. Nevertheless, the radicular cyst in deciduous dentition is an extremely rare condition with just under 4% among 1300 cases which were diagnosed as those cysts in both primary and permanent teeth (1). Generally, almost radicular cysts progress slowly and obtrusively. At the initial stage, radicular cysts usually present asymptomatic and tend to be overlooked, except detected by ordinary radiographic examination. The large lesions usually cause bony expansion, which presents a specific sign called “Ping-pong ball”. Besides, the marked mobility of the adjacent teeth, malocclusion, and displacement of underlying teeth may be found subsequently. Definitive diagnosis is established based on the clinical symptoms, X-ray findings, and histological characteristics. Treatment options include the combination of either cystic enucleation or marsupialization and removal of the involved primary tooth and conservation the succeeding permanent teeth.

Radicular cysts in primary teeth are frequently caused due to apical periodontitis which involves to dental caries (2). Others following dental trauma are less common and most occur in the incisors. Root canal treatment is indicated in cases of the irreversible pulpitis, necrotic pulpitis, and apical periodontitis. Grundy *et al.* (3) showed a number of such cases which were placed materials containing phenolic compounds into the root canals between appointments. Based on the published works, Takiguchi *et al.* (4) proposed the correlation between the rapid growth of such radicular cysts and some intracanal dressings. Materials for the endodontic obturation in primary teeth are required to be absorbable corresponding to the spontaneous absorption of these roots. Therefore, gutta-percha has been contraindicated to primary teeth, except in case of no succedaneous tooth (5).

## Case Report

A five-year-old girl was referred to the School of Odonto-Stomatology, Hanoi Medical University due to the appearance of a swelling on the left upper jaw since one and a half months ago. According to the patient’s mother, this abnormal enlargement was slightly growing in size and no evidence of neither pain nor pus discharge. The patient was taken to a dentist and prescribed with antibiotics but this lesion did not disappear. Based on the patient’s dental history, she had undergone a root canal therapy on the left maxillary primary first molar at a dental clinic one year before. The extra-oral observation denoted a diffused swelling on the left zygomatic region, which obliterated the left nasolabial fold and was larger than 3 cm in diameter. The lesion was firm in consistency and no tenderness to palpation.

The intra-oral examination indicated a hard bony mass on the labial surface spreading from the left maxillary primary canine to the second molar. T64 was restored with a large glass-ionomer cement filling and had slight mobility. The mucosa covering the swelling was pink in color and soft in consistency without any purulent drainage.

**Figure 1:**
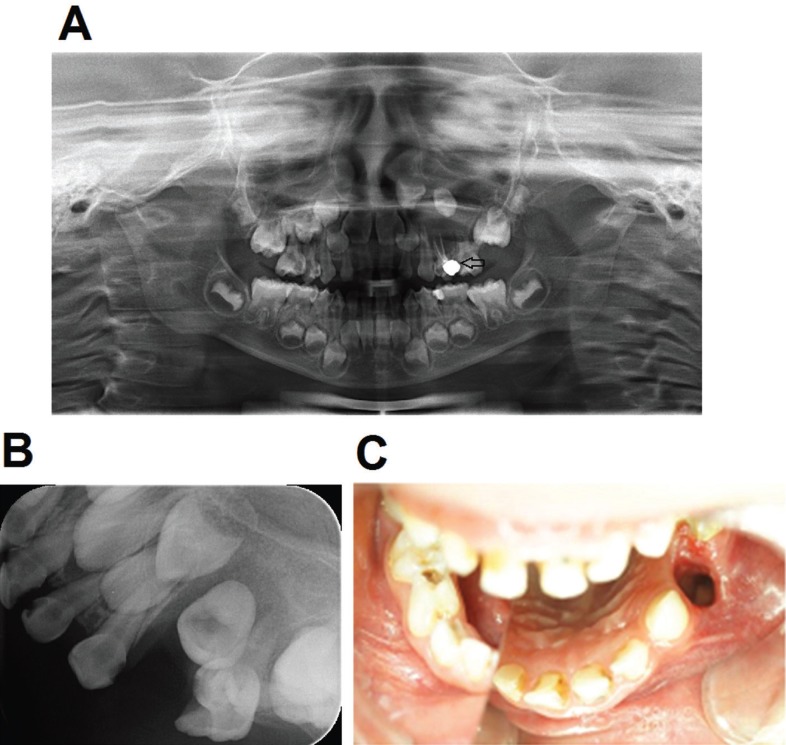
Preoperative and postoperative examination. The abnormal radiolucency also displaced upwards the bud of the left maxillary first premolar (A). The periapical radiograph showed a reduction in the size of the radiolucent lesion and a spontaneous eruption of T24 (B); and the marsupialization, the crown of T24 could be seen through the cyst window (C) 3 months after the procedure.

The panoramic radiograph illustrated a well-defined uniocular and oval-shaped radiolucency in the left maxillary region with a regular hyperostotic margin which extended from the distal surface of the root of T63 to the distal surface of T65, and surrounding totally the root of T64 without absorption of the root of T63 and T65 (Fig. 1A). Consequently, these features suggested a cystic lesion. The abnormal radiolucency also displaced upwards the bud of the left maxillary first premolar. After thorough analysis, we found both an absorption of the root of T64 and a radiolucent material in two root canals of this tooth. It was suspicious that T64 had been treated with gutta-percha filling.

Based on both of the patient’s symptoms and findings, we made a provisional diagnosis of RC correlated to the T64 which had been treated endodontically, and then indicated a cystic marsupialization treatment. The patient’s parents were explained thoroughly about their daughter’s condition and the pros and cons of the operation. Prior to the surgery, a written parental permission was made, routine blood tests were also performed and all indices were within normal limits. The operation was carried out under general anesthesia at the Hanoi Medical University Hospital. The antibiotics were preoperatively taken for prevention of infection.

**Figure 2:**
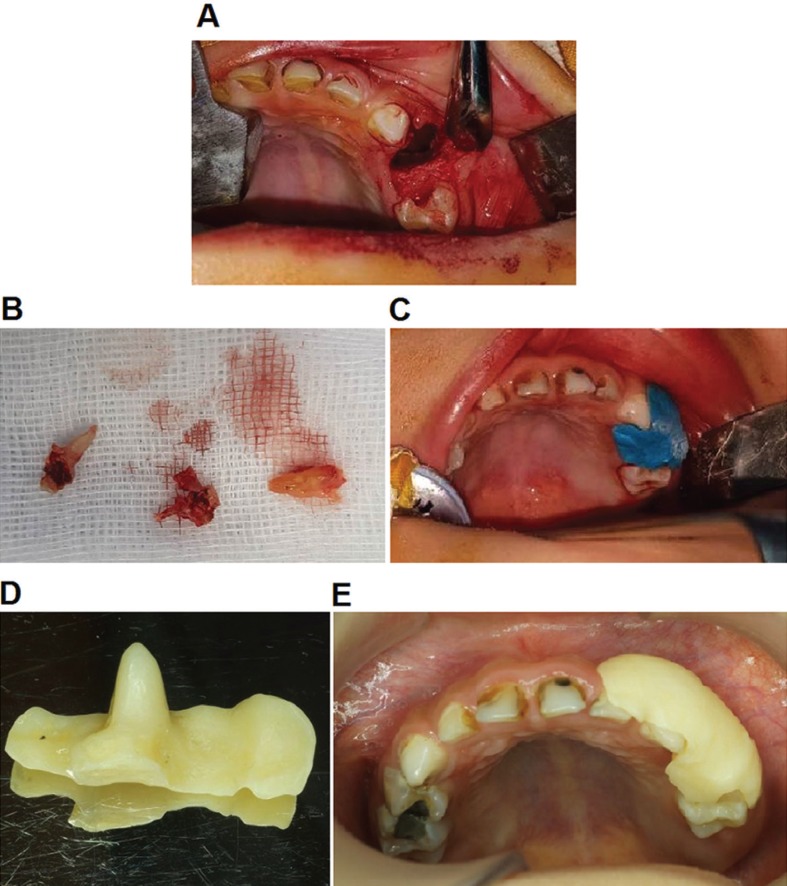
Marsupialization of the cystic cavity. Following T64 removal, the extraction socket was widened to make a cyst window and a biopsy sample was incised from the wall of the lesion for histopathological examination (A). It was clearly seen that there was gutta-percha sealer in both root canals of T64 (B). The impression of cyst opening was made with silicone before preparing the obturator (C). A customized acrylic obturator was prepared (D). 1 week after the procedure, the obturator was put into the cyst opening. Patient and her parents were carefully notified how to wear and remove the obturator (E).

The treatment plan included removal of the upper left primary first molar tooth and marsupialization of the RC in order to conserve the upper left first premolar tooth. Following T64 removal, the extraction socket was widened to make a cyst window and a biopsy sample was incised from the wall of the lesion for histological examination (Fig. 2A). The underlying cyst lining was thick, soft and looked like granulation tissue. It was clearly seen that there was gutta-percha sealer in both root canals of T64 (Fig. 2B). 500mg of Augmentin (Amoxicillin Clavulanate) and a Chlorhexidine gluconate 0.12% mouthwash were daily prescribed for one day before and ten days after surgery in order to avoid the postoperative contagiousness.

**Figure 3:**
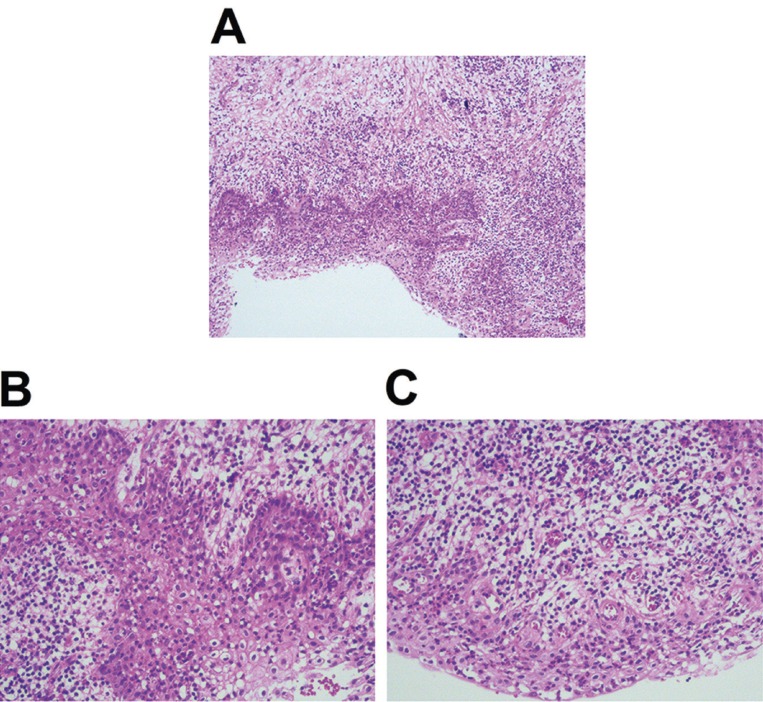
The histopathological examination of the cystic lesion. The histopathological examination of the lesion illustrated a cystic lumen lined with an inner non-keratinized stratified squamous epithelium with an outer connective tissue (A). There was an inflammatory cells infiltration in the non-keratinized stratified squamous epithelium (B). The proliferative fibro-vascular connective tissues with a significant infiltration of inflammatory cells consisting of the lymphocytes, plasma cells, macrophages and neutrophils (C).

The tissue sample was conserved in 10% formalin solution then stained with Hematoxyline and Eosin for digital histomorphometry analysis (Fig. 3A-C). The histological examination of the lesion illustrated a cystic lumen, which was lined with completely non-keratinized stratified squamous epithelium with some degenerated or ulcerated regions. The epithelium contained a multiple-cell layer thickness and the outer wall comprised proliferative fibro-vascular connective tissues with a significant infiltration of inflammatory cells consisting of the lymphocytes, plasma cells, macrophages and neutrophils (also called polymorphonuclear neutrophilic leukocytes). The needle-like cholesterol crystals were seen in the connective tissue wall. In summary, the histologic findings confirmed the final diagnosis as an RC in relation to an infectious primary tooth.

Subsequently, a customized acrylic obturator was prepared and put into the cyst window 1 week after the procedure (Fig. 2C-E). Patient and her parents were carefully notified how to wear and remove the obturator, and how to rinse the cyst cavity with saline daily. The patient had monthly appointments for follow-up and adjusting the denture corresponding to the size of the cyst opening. The resolution of the cystic lesion, as well as the eruption of the permanent successor, were assessed with a periapical radiograph in each recall. At 3 months after the procedure, periapical radiograph showed a reduction in the size of the radiolucent lesion and a spontaneous eruption of T24 (Fig. 1B). Moreover, the crown of T24 could be seen through the cyst window (Fig. 1C).

## Discussion

RCs are odontogenic cysts associated with an inflammatory condition caused by the non-vital or endodontically treated teeth. The invasion of bacteria from necrotic pulp to the periapical region stimulates the multiplication of ERM locating around the apex of the roots. It has been relatively infrequent to find out the RCs arising from primary teeth. According to a review done by Lustmann *et al.* (6), there were 28 such cases reported in the literature between 1898 and 1985. On a systemic review from 1927 to 2004, Nagata *et al.* (7) summarized 112 cases of radicular cysts deriving from primary dentition. This rare appearance in primary teeth may due to the following reasons:

• The time span of the deciduous dentition is shorter than that of the permanent dentition.

• A variety of accessory canals makes easy to drainage which decreases the cystic pressure and results in no symptoms (8).

• The amount of cells, which are capable of producing immune responses, in pulp tissues of primary teeth is considerably greater than that of permanent teeth (9). The distinction in biologic responses of pulp between the primary and permanent teeth may affect the growth of the radicular cyst.

• Additionally, radiolucent lesions around the apical area of a deciduous tooth may be diagnosed incorrectly or ignored or absorbed after extraction (8).

In general, radicular cysts develop progressively and represent asymptomatic, hence almost patients do not pay attention unless detected the abnormally radiolucent lesion with the routine dental radiography. Besides, the large cysts may produce expansion of the bone (presenting a specific sign called “Ping-pong ball”), displacement of the adjacent teeth and malocclusion. 23 in 28 cases on Lustmann’s research were observed and authors informed that: 1) the age of patients varied between 4 and 12 except one case at 19 years old, 2) the proportion of male to female was 1.6, 3) the lower molars were most common involved, 4) all cases caused by caries, 5) and all cases presented cystic lumen which was covered with non-keratinized stratified squamous epithelium in histological examination 6).

In this case, we decided the final diagnosis as a RC related to a primary tooth because of the following pieces of evidences: 1) the existence of a painless swelling and a bony expansion associated with a primary molar had endodontic treatment, 2) the existence of a well-defined radiolucent lesion surrounding the roots of a primary molar but not involving in the successive premolar, 3) histopathological features of a cystic lumen lined with non-keratinized stratified squamous epithelium, and 4) no relation between the cystic sheath and the successive permanent tooth during surgery. Marsupialization following extraction of the associated primary tooth aims to eliminate the growth of the cyst and preserve the permanent tooth. After 6-month follow up, the eruption of the permanent successor could be clearly seen through the cystic opening.

It could emphasize that the most popular etiology of radicular cyst in deciduous dentition is dental caries, while other possible cause is trauma but in lower frequency (10). Additionally, several researches reported the relation between the intracanal medicaments and the development of the radicular cysts in primary teeth. Takiguchi *et al.* (4) recommended a correlation between the intracanal dressing materials used for pulp treatment and the distinctive intraepithelial particles detected from cyst wall. Shetty *et al.* (10) demonstrated an update of 11 cases of radicular cysts involved in primary teeth with a half of them were suffered from pulp therapy and the rest caused by caries or trauma. Grundy *et al.* showed 17 cases of odontologic cysts related to primary molars following pulp therapy. Among these patients, the presence of the buccal expansion in those 10 cases and the dislocation of the corresponding permanent teeth in 12 others proposed that the materials used for pulp therapy (including phenol group which presented in cresol and parachlorophenol or formaldehyde) tend to promote the growth of the cysts (3). According to Deshpande A *et al.* (11), formaldehyde and its relative have been regularly used for pulp therapy. However, besides the bactericidal action, they may cause various unexpected effects including allergy and malignant potentiality, so these chemicals should be contraindicated in endodontic treatment. Sandhvarani B *et al.* (12) showed a case of bilateral radicular cysts involved in primary teeth with a history of endodontic treatment used zinc oxide eugenol, then suggested that cause of cysts could be an antigenic response to either the infection or the material filling. In such case, Savage NW *et al.* (13) described the histochemical image of distinctive intraepithelial inclusions containing an amorphous, eosinophilic material which indicated to include phenolic group. Studies on radicular cysts detected in deciduous teeth are listed in  Table 1.

**Table 1:**
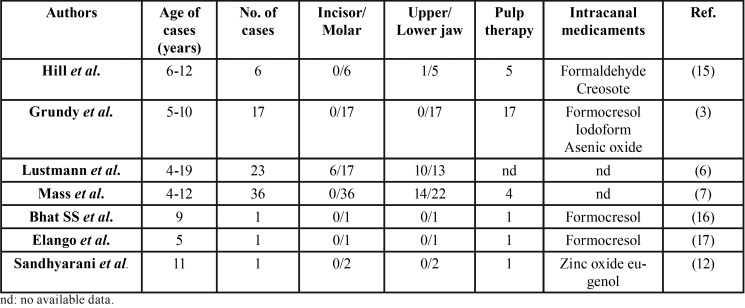
The cases of radicular cysts in primary teeth following root canal treatment.

Materials used for root canal obturation in primary teeth are required to be absorbable and do not prevent the eruption of the corresponding permanent teeth. Nowadays, according to guidelines of American Academy of Pediatric Dentistry (AAPD), various endodontic sealers for primary teeth have been recommended, for instance, Zinc oxide-eugenol paste, Iodoform and Calcium hydroxide. However, no material has been known as an ideal root canal sealer yet. Gutta-percha has been introduced to dentistry since the mid-19th century and known as the most common root canal filling for permanent teeth because it was obtained the Grossman’s standards of an optimal root canal filling material, which should be bacteriostatic without irritating to the periapical tissue. In contrast, gutta-percha has been contraindicated to primary teeth, except when there is no successive tooth. Ansari G and Mirkarimi (14) followed a case of gutta-percha root filling in the second primary molar with missing successor, after a long period time for follow up, it informed that using gutta-percha-filler could preserve the health and intact of those primary teeth. Up to now, numerous studies have reported about obturation of a retained deciduous tooth with gutta-percha. Otherwise, we have not found any document discussing the effect of gutta-percha to either formation or development of radicular cyst in primary dentition.

Together, several intracanal medicaments (including formocresol, iodoform, arsenic oxide, and their relatives) used for pulp therapy were considered as antigenic stimuli to the development of the radicular cyst and still no evidence indicated the relation between gutta-percha material filling and these lesions. In our case, as the primary molar was treated in a private dental clinic, no available evidence was obtainable about the material dressed in root canals except clear evidence of gutta-percha filling in the radiographic image and after surgery.

## Conclusions

The radicular cysts occurring in deciduous dentition are relatively rare and it is extremely important to early diagnose and appropriately treatment aiming to conserve the successive permanent teeth. In addition, it is necessary to have routine clinical and radiographic check-up for primary teeth had pulp treated.
